# Analysis of Human Cytomegalovirus-Encoded SUMO Targets and Temporal Regulation of SUMOylation of the Immediate-Early Proteins IE1 and IE2 during Infection

**DOI:** 10.1371/journal.pone.0103308

**Published:** 2014-07-22

**Authors:** Eui Tae Kim, Young-Eui Kim, Ye Ji Kim, Myoung Kyu Lee, Gary S. Hayward, Jin-Hyun Ahn

**Affiliations:** 1 Department of Molecular Cell Biology, Samsung Biomedical Research Institute, Sungkyunkwan University School of Medicine, Suwon, Republic of Korea; 2 Viral Oncology Program, The Sidney Kimmel Comprehensive Cancer Center, Johns Hopkins University School of Medicine, Baltimore, Maryland, United States of America; University of San Francisco, United States of America

## Abstract

Post-translational modification of proteins by members of the small ubiquitin-like modifier (SUMO) is involved in diverse cellular functions. Many viral proteins are SUMO targets and also interact with the cellular SUMOylation system. During human cytomegalovirus (HCMV) infection, the immediate-early (IE) proteins IE1 and IE2 are covalently modified by SUMO. IE2 SUMOylation promotes its transactivation activity, whereas the role of IE1 SUMOylation is not clear. We performed *in silico*, genome-wide analysis to identify possible SUMOylation sites in HCMV-encoded proteins and evaluated their modification using the *E. coli* SUMOylation system and *in vitro* assays. We found that only IE1 and IE2 are substantially modified by SUMO in *E. coli*, although US34A was also identified as a possible SUMO target *in vitro*. We also found that SUMOylation of IE1 and IE2 is temporally regulated during viral infection. Levels of SUMO-modified form of IE1 were increased during the early phase of infection, but decreased in the late phase when IE2 and its SUMO-modified forms were expressed at high levels. IE2 expression inhibited IE1 SUMOylation in cotransfection assays. As in IE2 SUMOylation, PIAS1, a SUMO E3 ligase, interacted with IE1 and enhanced IE1 SUMOylation. In *in vitro* assays, an IE2 fragment that lacked covalent and non-covalent SUMO attachment sites, but was sufficient for PIAS1 binding, effectively inhibited PIAS1-mediated SUMOylation of IE1, indicating that IE2 expression negatively regulates IE1 SUMOylation. We also found that the IE2-mediated downregulation of IE1 SUMOylation correlates with the IE1 activity to repress the promoter containing the interferon stimulated response elements. Taken together, our data demonstrate that IE1 and IE2 are the main viral SUMO targets in HCMV infection and that temporal regulation of their SUMOylation may be important in the progression of this infection.

## Introduction

Small ubiquitin-like modifier (SUMO) proteins are members of the ubiquitin-like protein family. Covalent modification of proteins by SUMO (SUMOylation) affects their activity, intracellular localization, stability, and interaction with other proteins and DNA. The cellular SUMOylation pathway, which is largely analogous to the ubiquitin modification pathway, regulates many important cellular processes [1], [2]. In brief, SUMO precursors are C-terminally processed to create an active form, which is activated by the formation of a thioester bond between the C-terminal glycine residue of SUMO and the active cysteine reside of a heterodimeric E1 activation enzyme, which comprises SAE1 and SAE2. SUMO is then transferred to the E2 conjugation enzyme, Ubc9, via an analogous thioester bond, and finally to the lysine residue of a substrate. SUMO E3 ligases, such as PIAS proteins, RanBP2, and Pc2, help transfer SUMO from Ubc9 to the substrate [3]–[5]. On most substrates, SUMO is conjugated to a lysine residue through an isopeptide linkage within the consensus sequence ΨKxE/D (where Ψ is a bulky hydrophobic residue and x is any amino acid), which is often found in the disordered region of proteins [6]–[9]. Both Ubc9 and the E3 ligases appear to control the substrate specificity of SUMOylation. SUMO can be released from a substrate through cleavage by proteases called SENP; therefore, SUMOylation is reversible [10]–[12]. Proteins also can interact with SUMO non-covalently through a SUMO-interacting motif (SIM), which is characterized by a stretch of hydrophobic residues, often flanked by acidic residues [13]–[16].

Evidence is accumulating that the cellular SUMOylation pathway plays a regulatory role in infection by many different viruses, including human cytomegalovirus (HCMV) [17], [18]. HCMV is an opportunistic pathogen that can cause congenital disease and produces serious disease complications in immunocompromised individuals. During the lytic cycle of HCMV infection, viral genes are expressed in a cascade fashion with immediate-early (IE), early, and late phases. The 72-kDa IE1 (also known as IE1-p71 or IE72) and 86-kDa IE2 (IE2-p86 or IE86) proteins are the major IE proteins that regulate activation of viral genes and modulate host cell functions [19]. Both IE1 and IE2 are modified by SUMO during HCMV infection.

IE2 is a strong transactivator that interacts with numerous cellular transactivators and is essential for early and late viral gene expression. IE2 is modified by SUMO at two lysine residues, K175 and K180. In transfection assays, SUMOylation of IE2 enhances the transactivation of diverse cellular and viral promoters by IE2 [20], [21]. Consistently, transactivation activity of IE2 has been correlated with its degree of SUMOylation [22]. IE2 directly binds to Ubc9 [20], [21] and PIAS1 [23]. Mutation of both K175 and K180 in a laboratory strain and a clinical isolate caused a modest decrease in virus replication, indicating that IE2 SUMOylation promotes the virus lytic cycle in the context of virus infection [24]. However, the effect of IE2 SUMOylation on viral growth appears to depend on the virus strains and infection conditions, since similar mutations in another laboratory strain did not significantly affect viral growth [25]. IE2 also non-covalently interacts with SUMO through a SIM adjacent to the SUMO conjugation sites. This SIM is necessary for efficient SUMOylation and transactivation activity of IE2, thereby promoting viral growth [24], [26]. The IE2 SIM promotes transactivation by IE2 by recruiting other SUMO-modified transcription cofactors, such as TAF12 [26].

IE1 is required for efficient viral gene expression, particularly at a low multiplicity of infection [27], [28]. IE1 also plays a key role in disarming host intrinsic and innate antiviral responses. IE1 disrupts PML nuclear bodies (NBs), also known as nuclear domain 10 (ND10) [29]–[32]. This activity correlates with the loss of SUMOylated PML NB components, such as PML and Sp100, which repress incoming viral genomes [33]–[35]. IE1 also interferes with type I interferon (IFN) signaling by directly targeting STAT2 using its near C-terminal region, and, to a lesser extent, by binding to STAT1 [36]–[38]. IE1 is modified by SUMO at K450 within the acidic domain [39], [40]. The role of IE1 SUMOylation in virus infection is unclear. IE1 SUMOylation has been reported to promote viral growth, while other studies have found a lack of significant impact [40]–[42]. We previously found that the SUMO-modified form of IE1 failed to interact with STAT2, suggesting that SUMOylation of IE1 may inhibit the ability of IE1 to downregulate type I IFN signaling [37]. The SUMOylation site of IE1 is close to its C-terminal chromatin-tethering domain; however, IE1 SUMOylation did not affect IE1 accumulation at mitotic chromosomes [43]. Phosphorylation of IE1 has been reported to decrease its SUMOylation [44].

In this study, we performed an *in silico* genome-wide analysis to identify HCMV-encoded SUMO targets. We found that viral IE1 and IE2 proteins might be the main SUMO targets. We also investigated whether SUMOylation of IE1 and IE2 is regulated during HCMV infection. Our results showed that high-level expression of IE2 and its SUMO-modified forms at the late stage of infection downregulates IE1 SUMOylation via competing PIAS1 binding, potentiating IE1 repression of interferon-stimulated gene (ISG) activation.

## Materials and Methods

### Plasmids

pSG5 [45]-based expression plasmids for IE1 (pJHA303), IE2 (pJHA124), GST-IE2(346–542) (pHJK13), flag-SUMO-1 (pJHA312), and flag-SUMO-2 (pJHA342) were previously described [21], [23], [42]. Plasmids for HA-IE1 (pDJK170), HA-UL53 (pMK56), and GST-IE1 (pDJK175) were produced by moving the cDNAs from pENTR vectors (Invitrogen) to pSG5-HA and pGEX-3-based destination vectors, respectively, using LR Clonase (Invitrogen). Similarly, plasmid for His-IE1 (pSHJ9) was produced with the pDEST17 (with a 6His tag) destination vector (Invitrogen), and plasmids for HA-PIAS1 (pHJK1), SRT-PIAS1 (pSAN22), myc-PIAS1 (RYK595), and myc-IE2(346–542) (pRYK593) were produced with the pSG5-HA, pSG5-SRT or pCS3-MT (with a 6myc tag) [46]-based destination vectors. pCMV-Flag-PIAS1 was kindly provided by Ke Shuai (UCLA, Los Angeles, CA, USA). Plasmids for GST-SAE2/SAE1, in which GST-SAE2 and SAE1 are translationally linked via a ribosome binding site, His-Ubc9, and GST-SUMO-1_GG_ were previously described [47], and the plasmid for His-SUMO-1_GG_ was produced with the pDEST17 destination vector using LR Clonase. pT-E1E2S1, which encodes the E1 and E2 enzymes for SUMO conjugation as well as the active form of SUMO-1 [48], was used to introduce a synthetic SUMO-1 conjugation pathway into *E. coli*.

### Cloning HCMV ORFs

HCMV open reading frames (ORFs) were cloned as previously described [49]. HCMV ORFs were PCR amplified using primers based on the GenBank sequences AY446894, GU937742, and FJ616285. Bacterial artificial chromosomes Toledo-BAC [50] and Towne-BAC [51] were used as templates (gifts from H. Zhu, UMDNJ-New Jersey Medical School, Newark, New Jersey, USA). The 5′ primers contained the attB1 recombination site, and the 3′ primers contained the attB2 recombination site (attB1, 5′-GGGGACAAGTTTGTACAAAAAAGCAGGCTCC-3′; attB2, 5′-GGGGACCACTTTGTACAAGAAAGCTGGGTC-3′) (Invitrogen). Some long ORFs were amplified in segments. For some ORFs that encode spliced products, cDNAs prepared form virus-infected cells were used for PCR amplification. PCR products of the correct size were recombined into the gateway vector pDONR201 (to make pENTR clones) using BP Clonase (Invitrogen). *E. coli* that had been transformed with the reaction products (pENTR clones) were selected, and the DNA inserts were analyzed by digestion with BsrGI and sequencing. Yeast cells expressing plasmids encoding GAL4-activation domain (AD)-ORF fusions were produced by transferring the ORFs from pENTR vectors to a pACTII [21]-based destination vector using LR Clonase.

### Transfection

293T cells were transfected via the *N*,*N*-bis-(2-hydroxyethyl)-2-aminoethanesulfonic acid-buffered saline (BBS) version of the calcium phosphate method, as described previously [23].

### Immunoblot analysis

Samples were prepared by boiling in loading buffer, separated by SDS-PAGE, and transferred to a nitrocellulose membrane (Schleicher & Schuell, Dassel, Germany). The membrane was blocked for 1 h in PBS-T [PBS plus 0.1% Tween-20 (Sigma)] containing 5% skim milk and then washed with PBS-T. After incubation with the appropriate antibody, the proteins were visualized by the standard procedure using an enhanced chemiluminescence system (Roche). For SUMOylation assays in transfected cells, cells were washed with PBS containing 5 mM NEM, and the samples were prepared by boiling in SDS loading buffer.

### Coimmunoprecipitation (CoIP) assays

293T (8×10^5^ in 100-mm dish) cells were harvested and sonicated in 1 ml CoIP buffer (50 mM Tris-Cl [pH 7.4], 50 mM NaF, 5 mM sodium phosphate, 0.1% Triton X-100, containing protease inhibitors [Sigma]) using a microtip probe (Vibra cell; Sonics and Materials, Inc., USA) for 10 sec (pulse on: l sec, pulse off: 3 sec). Clarified cell lysates were incubated for 16 h with appropriate antibodies at 4°C. Thirty microliters of a 50% slurry of protein A- and G-Sepharose (Amersham) was then added. After a 2 h incubation at 4°C, the mixture was pelleted and washed several times with CoIP buffer. The beads were resuspended and boiled for 5 min in loading buffer. Each sample was analyzed by SDS-PAGE, and immunoblotting was performed.

### *In vitro* binding assays with GST fusion proteins

The GST and GST-IE1 fusion proteins were prepared in *E. coli* by standard procedures. The [^35^S]Met-labeled PIAS1 was produced from a pSG5-derived template using the TNT Quick Coupled Transcription/Translation System (Promega) as specified by the manufacturer. The standard procedure for the GST pull-down assays was described previously [21].

### *In vitro* SUMOylation assays

Recombinant GST fusion proteins were expressed in *E. coli*, and purified on glutathione-agarose 4B (Peptron) according to the manufacturer's instructions. His-tagged proteins were also produced in *E. coli* and purified on Ni-NTA beads (Invitrogen) according to the manufacturer's guidelines. Typical SUMOylation reactions were conducted in a 30 µl volume containing 70 nM GST-SAE2/SAE1, 1 µM His-Ubc9, and 9 µM His-SUMO-1_GG_ or GST-SUMO-1_GG_ in buffer (50 mM Tris-HCl [pH 7.5], 10 mM MgCl_2_, 1 mM DTT, and 5 mM ATP). To prepare flag-PIAS1 protein, 293T cells in a 150-mm dish were transfected with 30 µg of flag-PIAS1-expressing plasmid, followed by immunoprecipitation of total cell lysates with 50 µl of anti-flag M2 antibody. SUMOylation reaction mixes were incubated for 1 h at 37°C. After terminating the reaction with SDS sample buffer containing β-mercaptoethanol, the reaction products were fractionated by SDS-PAGE.

### Antibodies

Anti-His (H-3) mouse monoclonal antibody (MAb) conjugated with horseradish peroxidase (HRP) and anti-GST MAb (B-14) were purchased from Santa Cruz. Anti-HA rat MAb (3F10) and anti-myc mouse MAb (9E10) conjugated with HRP were purchased from Roche. Anti-flag mouse MAb M2 was obtained from Sigma. Anti-IE1 polyclonal antibody (PAb) was raised in rabbits using the purified IE1 protein. Mouse MAb 8131, which detects epitopes present in both IE1 and IE2 (exons-2 and -3), was purchased from Chemicon (Temecula, CA, USA). Mouse MAbs specific for IE1 (6E1) and IE2 (12E2) were purchased from Vancouver Biotech and mouse MAb against β-actin was purchased from Sigma. Mouse MAb against SRT epitope was previously described [23].

### Luciferase reporter assay

Cells were collected and lysed by three freeze-thaw steps in 200 µl of 0.25 M Tris-HCl (pH 7.9) plus 1 mM dithiothreitol. Cells extracts were clarified in a microcentrifuge and 30 µl of extracts were incubated with 350 µl of reaction buffer A (25 mM glycyl-glycine [pH 7.8], 15 mM ATP, and 4 mM EGTA) and then mixed with 100 µl of 0.25 mM luciferin (Sigma-Aldrich) in reaction buffer A. A TD-20/20 luminometer (Turner Designs) was used for a 10-s assay of the photons produced (measured in relative light units).

## Results

### *In silico* analysis of SUMOylation sites in HCMV-encoded proteins and evaluation of SUMOylation

To identify SUMO targets in the entire HCMV genome, we used the SUMOplot Analysis Program (http://www.abgent.com/sumoplot) and SUMOsp program (http://sumosp.biocuckoo.org/online.php) [52] to predict and score SUMO modification sites in proteins. We tested 165 HCMV ORFs from the HCMV Towne and Toledo strains. From this *in silico* analysis, 24 ORFs, including UL122 (IE2) and UL123 (IE1), which were previously identified as SUMO targets, were predicted with high probability to contain SUMO modification sites by both programs (Table 1).

**Table 1:** HCMV ORFs that contain possible SUMOylation sites.

ORF^a^	Template	Amino acids	Lys position	Consensus sequence	SUMOsp score	SUMOplot score	pEXP-GST clone	Lys position (in Merlin ORF)
US34A	Towne-BAC	64	38	VKQE	5.18	0.93	pETK283	38
UL123(Ex2/3/4)	Towne cDNA	471	450	VKSE	4.95	0.93	pDJK175	450
UL122(Ex2/3/5)	Towne cDNA	579	175, 180	IKQE,IKPE	3.97, 3.32	0.94, 0.94	pDJK183	175, 180
UL150	Toledo cDNA	640	124	AKSD	3.59	0.79	pETK279	124
UL84	Towne-BAC	587	73, 163, 464	LKTP,KKKE,LKMP	3.40, 3.39, 3.70	0.8, 0.48, 0.8	pETK271	73, 163, 464
UL35	Towne-BAC	640	137	VKPE	2.18	0.93	pETK263	137
RL10	Towne-BAC	168	35	VKAE	2.13	0.93	pETK259	35
UL27	Towne-BAC	608	431, 496	FKGE,IKRE	0.52, 2.2	0.85, 0.94	pETK262	431, 496
UL46	Towne-BAC	290	27, 159	AKRE,LKTE	1.11, 2.05	0.79, 0.91	pETK265	27, 159
UL89B(Ex2)^b^	Towne-BAC	378	19	IKKE	2.04	0.94	pETK272	19
UL111A(Ex1/2/3)	Towne cDNA	176	108	LKTE	1.94	0.91	pETK276	108
UL83	Towne-BAC	561	457	LKAE	1.69	0.91	pETK270	457
UL57	Towne-BAC	1235	417	LKDE	1.64	0.91	pETK268	417
US27	Towne-BAC	364	320	VKQE	1.54	0.93	pETK282	320
UL94	Towne-BAC	345	324	VKVE	1.46	0.93	pETK273	324
UL98	Towne-BAC	584	214	IKHE	1.41	0.94	pETK274	214
UL72	Towne-BAC	388	35	MKEE	1.34	0.8	pETK269	35
UL105	Towne-BAC	956	29, 377	AKIE,LKEE	0.99, 1.30	0.79, 0.91	pETK275	29, 377
UL48	Towne-BAC	2240	604, 1032	AKQE,VKGE	1, 1.01	0.79, 0.93	pSE45	605, 1033
UL54	Towne-BAC	1242	947	VKLE	0.95	0.93	pETK267	947
UL43	Towne-BAC	423	278	MKRE	0.94	0.8	pETK264	278
UL49	Towne-BAC	470	359	LKCE	0.77	0.91	pETK266	359
UL44	Towne-BAC	433	410	AKEE	0.7	0.79	pRYK18	410
UL148	Towne-BAC	316	275	AKAE	0.67	0.79	pETK278	275

a ORFs are listed by score with the SUMOsp program.

b UL89 exon 2. For the full-length UL89 protein, one lysine residue (K315) was predicted as a SUMO attachment site with the same score from both SUMOplot and SUMOsp.

**Figure 1:**
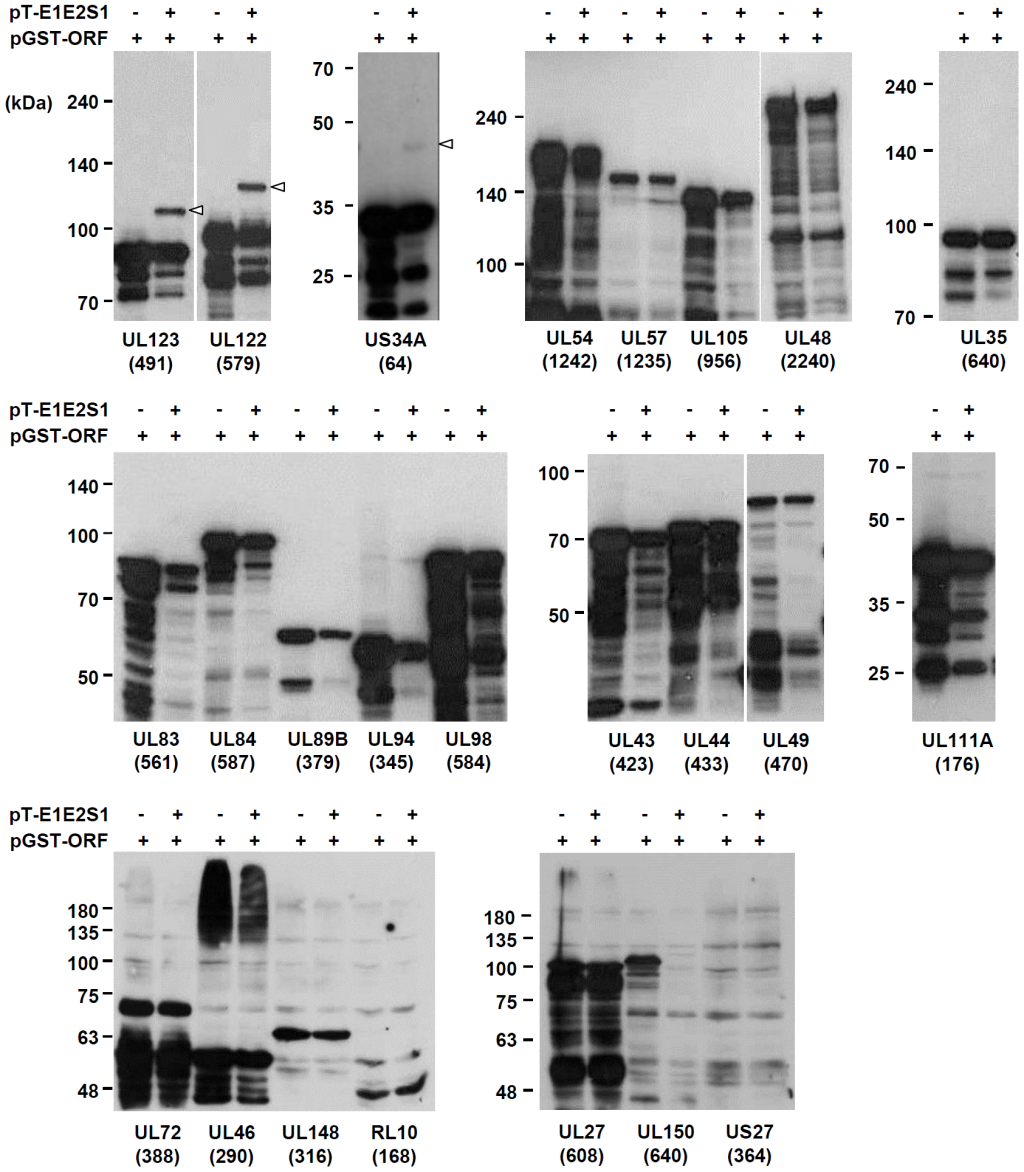
SUMOylation analysis of HCMV proteins in bacteria. *E. coli* (BL21) cells were transformed with plasmids expressing GST-HCMV ORF (ampicillin-resistant) or cotransformed with plasmids expressing GST-HCMV ORF and pT-E1E2S1 (chloramphenicol-resistant). One milliliter of bacterial cell culture was induced with 0.4 mM IPTG for 5 h at 30°C. Total cell lysates were prepared by boiling the cell pellet in 200 µl of 1× protein loading dye. Clarified cell lysates were separated by SDS-PAGE, and immunoblot analysis was performed with anti-GST antibody. HCMV ORFs fused to GST and the ORF sizes (number of amino acids in parenthesis) are indicated. The SUMO-modified forms of UL123 (IE1), UL122 (IE2), and US34A are indicated as open arrowheads.

We next investigated whether the predicted 24 proteins are covalently modified by SUMO. The HCMV ORF library was produced in the pENTR vector (Invitrogen) and pGEX-3-derived plasmids expressing GST-ORF fusion proteins were produced (Table 1) (see Material and Methods). *E. coli* BL21 cells were transformed with pGST-ORF or cotransformed with pGST-ORF and pT-E1E2S1, which encodes a SAE2/SAE1 fusion (E1), Ubc9 (E2), and SUMO-1_GG_, an active form of SUMO-1. After the cells were grown, expression of GST-fusion proteins was induced with IPTG, and total cell lysates were prepared and immunoblotted with anti-GST antibody. The results showed that UL123 (IE2) and UL122 (IE1) were substantially modified by SUMO, and US34A was weakly modified by SUMO in this *E. coli* SUMOylation system (Fig. 1). We could not detect SUMOylated bands for 19 proteins (UL84, UL35, RL10, UL27, UL46, UL89 exon 2, UL111A, UL83, UL57, UL27, UL98, UL72, UL105, UL48, UL54, UL43, UL49, UL44, and UL148) in these assays (Fig. 1). We could not evaluate SUMOylation of UL150 and US27 in *E. coli*, since GST-UL150 became undetectable in *E. coli* cells that received both pGST-UL150 and pT-E1E2S1 probably due to change of protein stability, and GST-US27 was not expressed or expressed as several week bands, making detection of SUMOylated forms difficult (Fig. 1).

**Figure 2:**
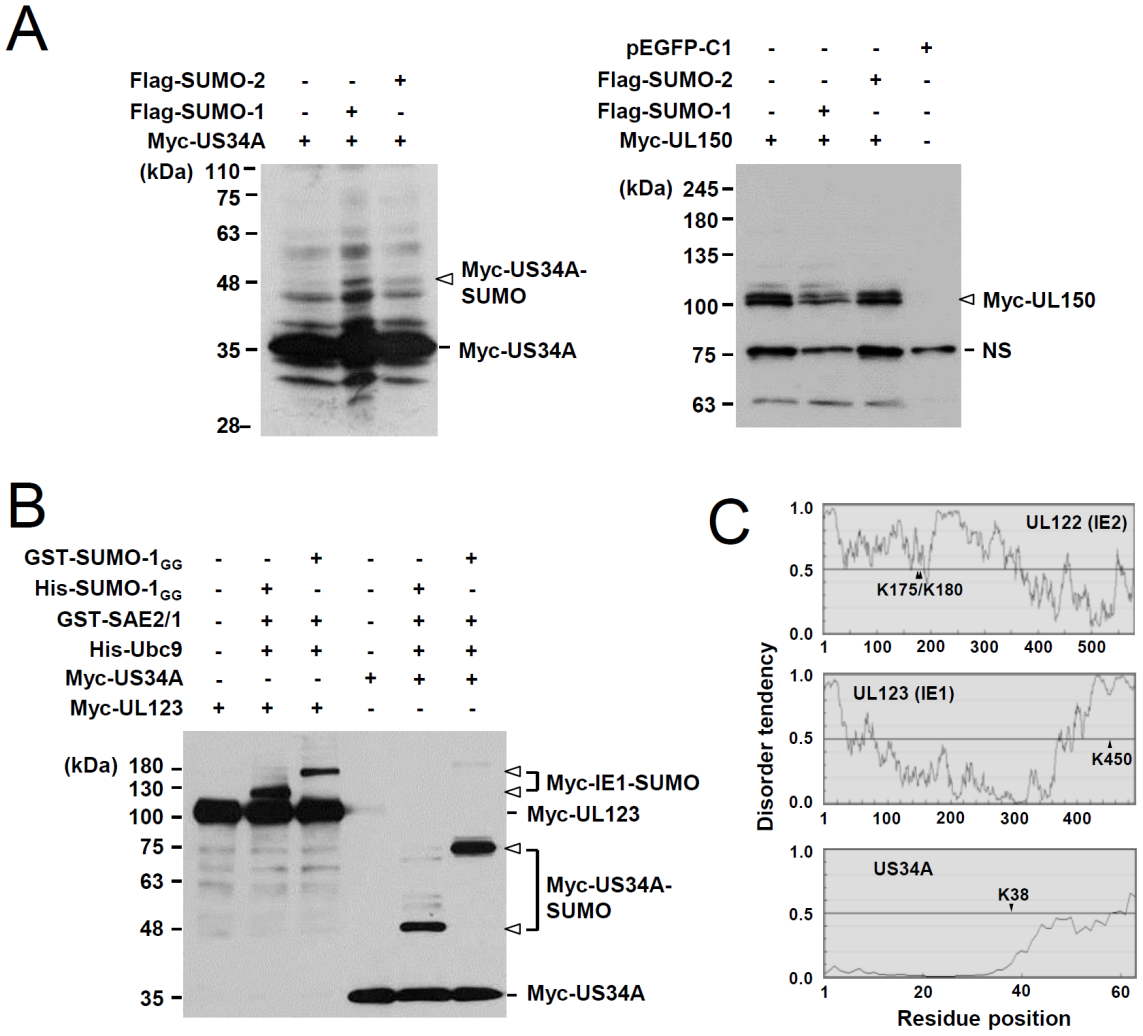
Evaluation of SUMOylation in cotransfection and *in vitro* assays. (A) 293T cells in six-well plates were cotransfected with 0.5 µg of plasmid expressing myc-US34A, myc-UL150, flag-SUMO-1, or flag-SUMO-2 as indicated. At 48 h, total cell lysates were prepared and immunoblotted with an anti-myc antibody. The bands corresponding to unmodified and SUMO-modified forms of myc-US34A and unmodified myc-UL150 are indicated. NS, non-specific bands. (B) *In vitro* SUMOylation reactions. Myc-UL123(IE1) and myc-US34A produced by *in vitro* transcription/translation were incubated with GST-SAE2/1, His-Ubc9, and His-SUMO-1_GG_ or GST-SUMO-1_GG_ as indicated. The reaction products were analyzed by SDS-PAGE (8%) and immunoblot assays with a myc-IE1 antibody. Unmodified and SUMO-modified forms of IE1 and US34A are indicated. (C) The disorder in UL122 (IE2), UL123 (IE1), and US34A was predicted with the IUPred program (http://iupred.enzim.hu). The lysine residues modified by SUMO (for IE1 and IE2) or predicted to be SUMOylation sites (for US34A) are indicated.

We further tested SUMOylation of US34A, UL150, and US27 using cotransfection assays. 293T cells were cotransfected with plasmids expressing a viral protein and SUMO-1, and immunoblotting was performed. We detected a small amount of SUMOylated US34A, but did not detect SUMOylated UL150 (Fig. 2A). US27 SUMOylation could not be evaluated because US27 migrated as a smear in cotransfected cells (data not shown), as previously described [53], [54]. US34A SUMOylation was further investigated *in vitro* using purified bacterial GST-SAE2/SAE1 (E1), His-Ubc9 (E2), and His- or GST-tagged SUMO-1_GG_, an active form of SUMO-1. The results showed that US34A was modified by SUMO-1 as efficiently as UL123 (IE1) *in vitro*, suggesting that US34A may be another SUMO target encoded by HCMV (Fig. 2B). However, unlike UL122 (IE2) and UL123 (IE1), the region of US34A containing the predicted SUMOylation site, K38, did not have a tendency to be highly disordered (Fig. 2C). Overall, our *in silico* genome-wide analysis of HCMV-encoded SUMO targets and subsequent cotransfection and *in vitro* assays demonstrated that IE1 and IE2 might be main SUMO targets in HCMV. These experiments also suggested that UL34A may be a potential SUMO target.

### SUMOylation patterns of IE1 and IE2 during HCMV infection

**Figure 3:**
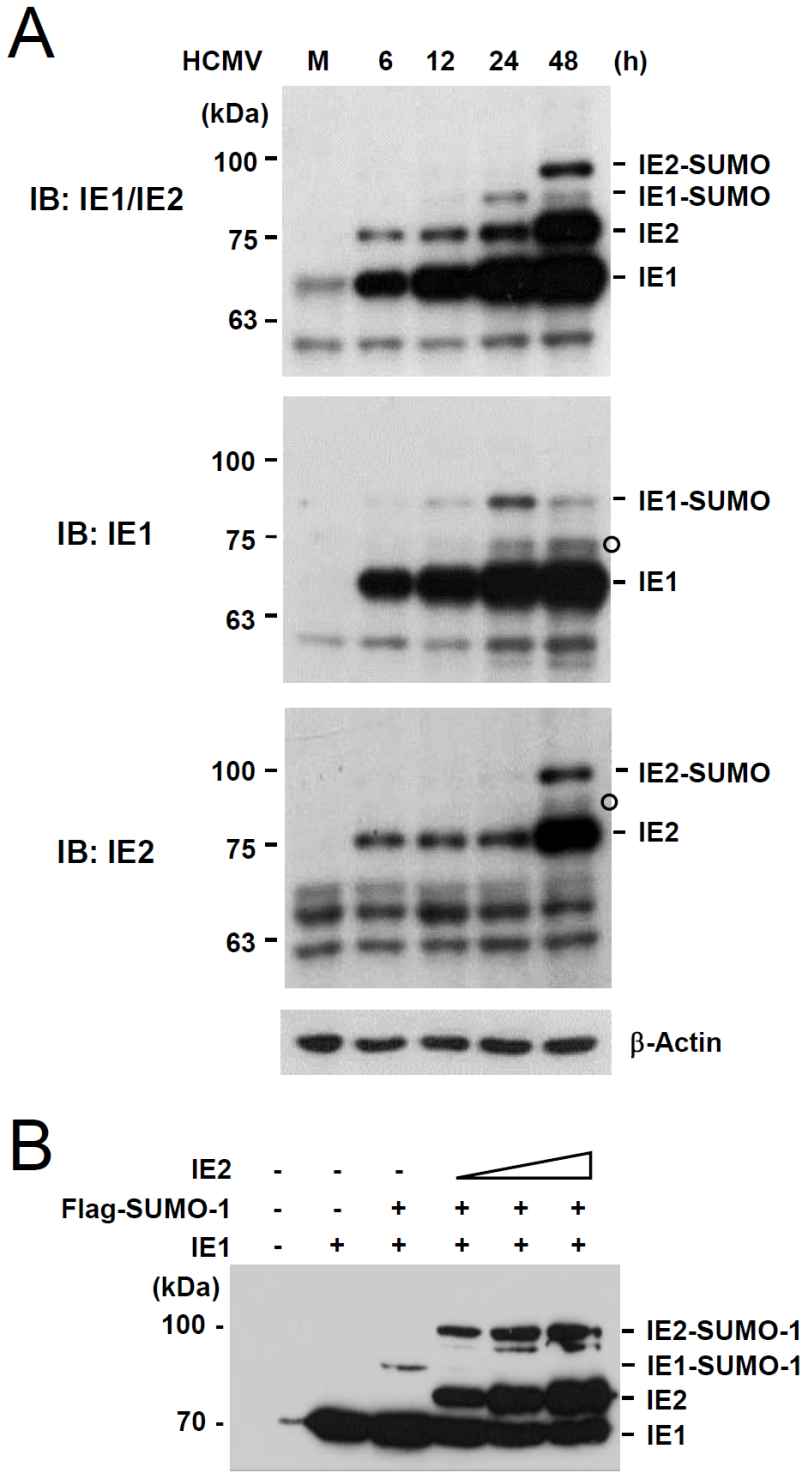
SUMOylation patterns of IE1 and IE2 during HCMV infection. (A) HF cells were mock-infected or infected with HCMV at an MOI of 5. Total cell lysates were prepared at indicated time points and immunoblotting was performed with antibodies that recognize IE1 (6E1), IE2 (12E2), or both IE1 and IE2 (8131). The β-actin levels are shown as a loading control. The bands indicated as open circles appear to be non-specific or represent other modified forms of IE1 and IE2. (B) 293T cells in six-well plates were cotransfected with plasmids expressing IE1 (1 µg), flag-SUMO-1 (1 µg), and increasing amounts of IE2 (0.3, 1, and 3 µg), as indicated. At 48 h, total cell lysates were prepared and immunoblot assays were performed with anti-IE1/IE2 antibody.

We next investigated the change in SUMOylation patterns of IE1 and IE2 during HCMV infection. Total cell lysates prepared at different time points after HCMV infection were immunoblotted with antibodies specific for IE1, IE2, or both. We found that IE1 SUMOylation peaked 24 h after infection and then declined at 48 h when the level of IE2 and its SUMOylation was drastically increasing (Fig. 3A). This result suggested that IE1 SUMOylation is temporally regulated during virus infection and that this change depends on the IE2 level. The effect of IE2 expression on IE1 SUMOylation was further examined in cotransfection assays. Immunoblots showed that the level of SUMOylated IE1 was reduced when IE2 was overexpressed, suggesting an inhibitory effect of IE2 on IE1 SUMOylation (Fig. 3B).

### PIAS1 interacts with IE1 and acts as a SUMO E3 ligase

**Figure 4:**
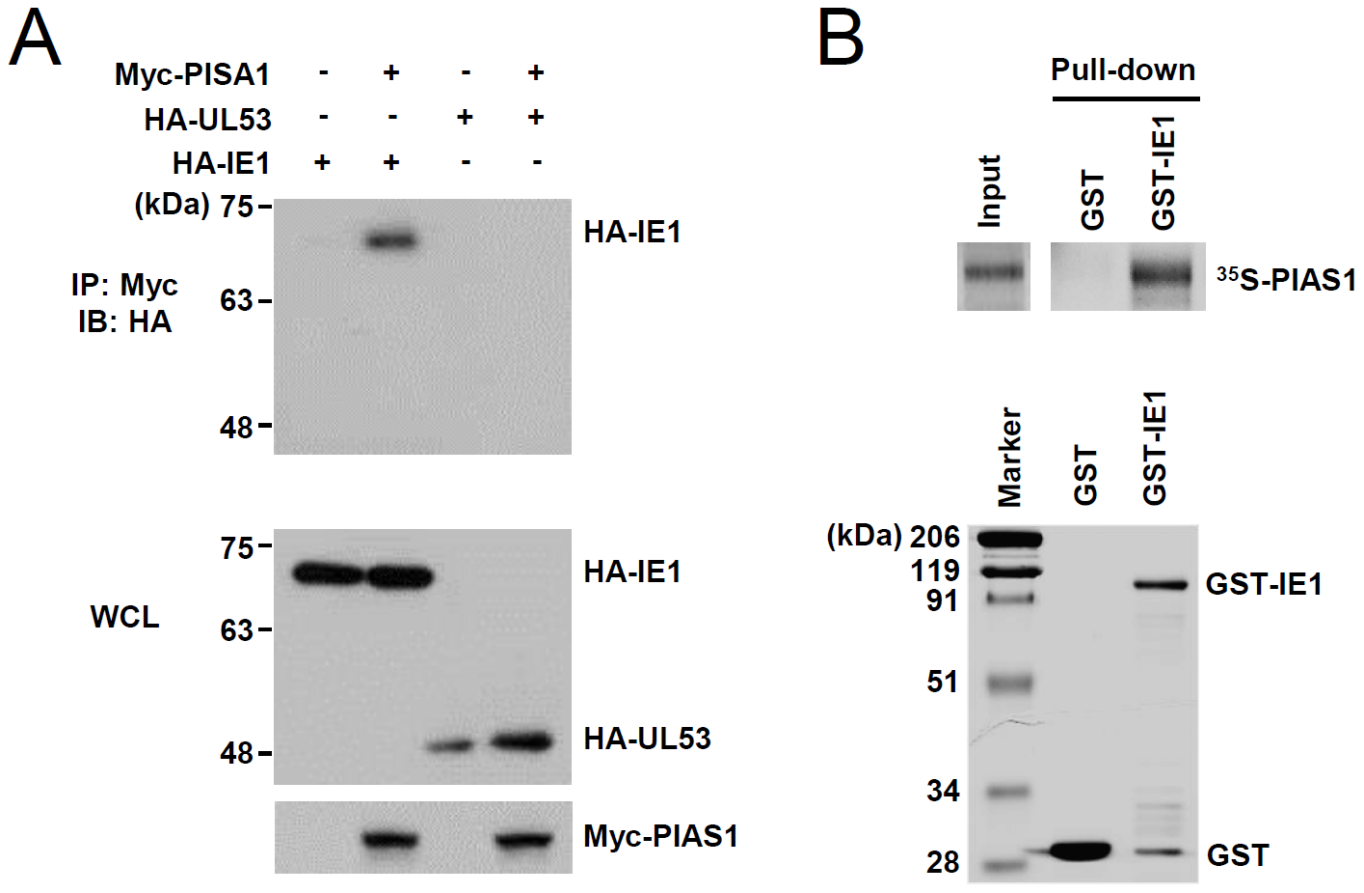
Interaction of IE1 with PIAS1. (A) 293T cells in a 100-mm dish were cotransfected with 5 µg of plasmids expressing myc-PIAS1 and HA-IE1 or HA-UL53, as indicated. At 48 h, total cell lysates were prepared and immunoprecipitated with an anti-myc antibody, followed by immunoblotting with an anti-HA antibody. The levels of HA-IE1, HA-UL53, and myc-PIAS1 in whole cell lysates (WCL) were also shown by immunoblotting. (B) The GST and GST-IE1 proteins purified from bacteria were used in GST pull-down assays. Five micrograms of GST and GST-IE1 proteins were immobilized on glutathione-Sepharose beads and were incubated with *in vitro*-translated and [^35^S]-methionine–labeled PIAS1. Input PIAS1 (5%) and the GST pull-down samples were separated by SDS-PAGE and visualized by autoradiography (upper panels). The purified GST and GST-IE1 used in pull-down assays are shown by SDS-PAGE and Coomassie Brilliant Blue staining (lower panel).

We hypothesized that increased IE2 expression might compete with IE1 for the cellular SUMOylation machinery. To address this question, we first tested whether IE1 SUMOylation requires PIAS1, a SUMO E3 ligase that acts as an E3 for IE2 SUMOylation [23]. In cotransfection assays, PIAS1 was coimmunoprecipitated with IE1 but not with UL53 (a negative control), suggesting that PIAS1 specifically interacts with IE1 (Fig. 4A). Furthermore, in GST pull-down assays, bacterial GST-IE1protein effectively interacted with PIAS1 produced by *in vitro* transcription/translation (Fig. 4B). These results indicated that IE1 indeed interacts with PIAS1.

**Figure 5:**
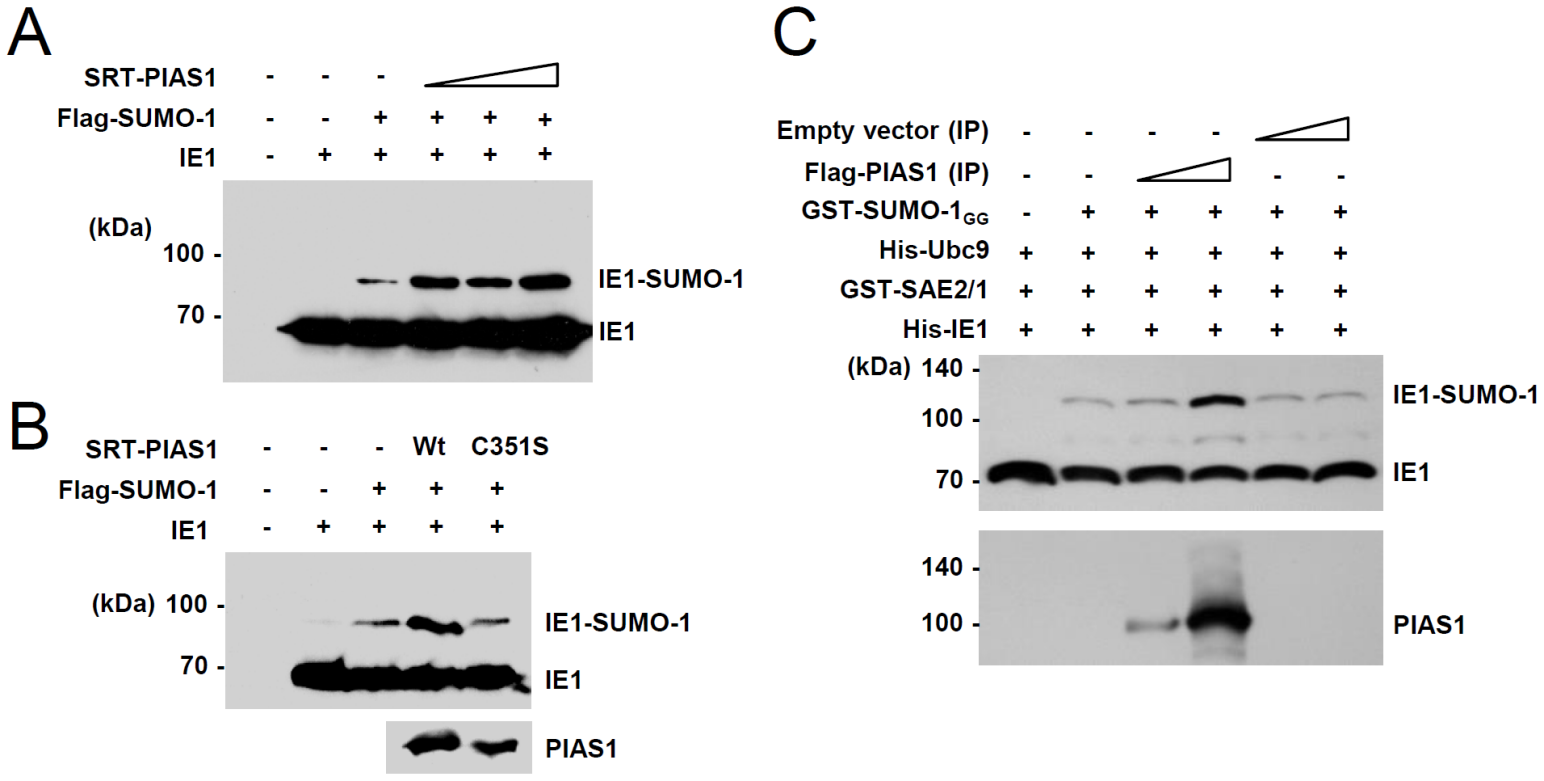
Enhancement of IE1 SUMOylation by PIAS1. (A) 293T cells in six-well plates were cotransfected with plasmids expressing IE1 (1 µg), flag-SUMO-1 (1 µg), and increasing amounts of SRT-PIAS1 (0.3, 1, and 3 µg), as indicated. At 48 h, total cell lysates were prepared and immunoblotted with an anti-IE1 antibody. (B) 293T cells were cotransfected with plasmids expressing IE1 (1 µg), flag-SUMO-1 (1 µg), and wild-type or C351S mutant SRT-PIAS1 (0.5 µg), as indicated. At 48 h, total cell lysates were prepared and immunoblotted with anti-IE1 or anti-SRT antibodies. (C) *In vitro* SUMOylation reactions were conducted with bacterially purified His-IE1, GST-SAE2/1, His-Ubc9, and GST-SUMO-1_GG_ proteins, and immunoprecipitated flag-PIAS1 proteins (see Materials and Methods). The reaction products were analyzed by SDS-PAGE (8%) and immunoblot assays with anti-IE1 antibody. The amounts of flag-PIAS1 protein used were also shown by immunoblotting with anti-flag antibody.

We next tested whether PIAS1 enhances IE1 SUMOylation. In cotransfection assays, PIAS1 increased SUMOylation of IE1 in a dose-dependent manner (Fig. 5A). A catalytically inactive PIAS1 mutant (C351S), in which the active site cysteine at amino acid 351 was replaced with serine [23], did not increase IE1 SUMOylation. This result suggests that PIAS1 may act as an E3 ligase for IE1 SUMOylation (Fig. 5B). To confirm the role of PIAS1 in IE1 SUMOylation, we performed *in vitro* SUMOylation assays. We used PIAS1 protein that was immunoprecipitated from transfected cells, because PIAS1 is not easy to produce in a soluble fraction in *E. coli*. Consistent with the results of cotransfection assays, we found that immunoprecipitated PIAS1 increased IE1 SUMOylation in a dose-dependent manner *in vitro*. These data indicate that PIAS1 acts as a SUMO E3 ligase for IE1 SUMOylation (Fig. 5C).

### IE2 inhibits PIAS1-mediated SUMOylation of IE1

**Figure 6:**
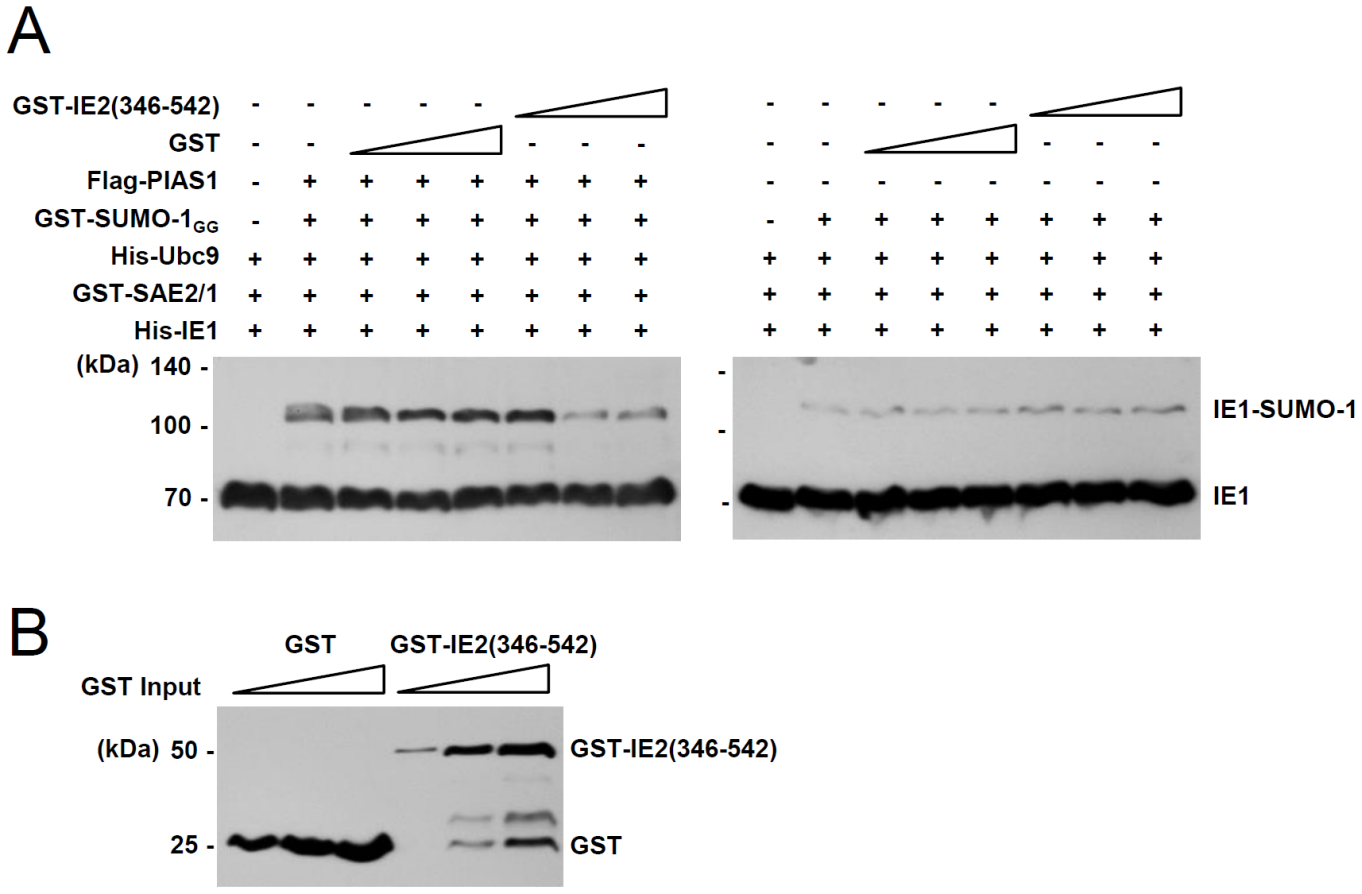
Inhibition of the PIAS1-mediated IE1 SUMOylation by IE2 *in vitro*. (A) *In vitro* SUMOylation reactions for IE1 were conducted as in Fig. 5C using His-IE1, GST-SAE2/1, His-Ubc9, GST-SUMO-1_GG_, and increasing amounts (0.1, 0.5, and 1 µg) of GST or GST-IE2(346–542) with (left panel) or without (right panel) immunoprecipitated flag-PIAS1. The reaction products were analyzed by SDS-PAGE (8%) and immunoblot assays with anti-IE1 antibody. (B) The GST and GST-IE2(346–542) proteins added to *in vitro* SUMOylation reactions were detected by immunoblotting with an anti-GST antibody.

To address whether IE2 competes with IE1 for PIAS1 in SUMOylation reactions, we examined the effect of the IE2(346–542) fragment on IE1 SUMOylation *in vitro.* IE2(346–542) contains the PIAS1 binding region [23], but not sites for covalent or non-covalent SUMO attachment [26]. The *in vitro* SUMOylation assays showed that the level of IE1 SUMOylation produced in reactions containing SAE2/SAE1 (E1) and Ubc9 was increased in the presence of PIAS1, but this PIAS1-mediated IE1 SUMOylation was inhibited by IE2(346–542) (Fig. 6). In a control experiment, IE1 SUMOylation without PIAS1 was not affected by IE2(346–542) (Fig. 6). These results demonstrate that IE1 SUMOylation was negatively regulated by the PIAS1-binding activity of IE2. The moderate inhibitory effect of IE2(346–542) on IE2 SUMOylation was also observed in cotransfection assays (data not shown).

**Figure 7:**
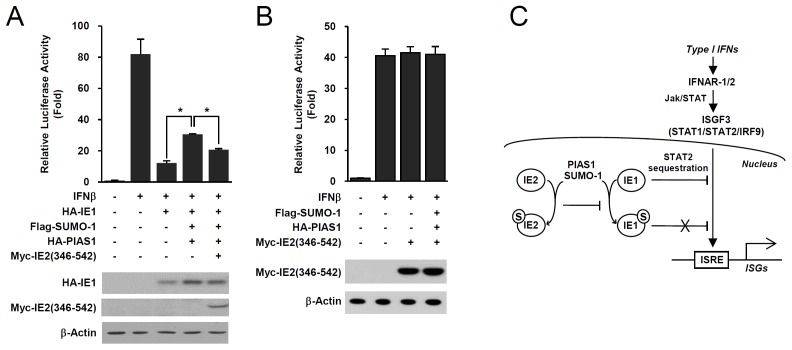
IE2 reverses the SUMOylation-dependent inhibition of IE1 activity to downregulate ISRE activation. (A and B) The reporter assays using the ISG54 ISRE-luciferase construct. 293T cells in 12-well plates were cotransfected with 0.5 µg of the ISG54 ISRE-luciferase reporter construct and plasmids expressing HA-IE1, flag-SUMO-1, HA-PIAS1, and myc-IE2(346–542) as indicated. The total amount of plasmid was adjusted with empty vectors. At 24 h, cells were untreated or treated with IFNβ (1,000 units/ml) for 8 h, and luciferase reporter assays were performed. The results shown are the mean values and standard errors of three independent experiments. Statistical significance between samples was determined using Student's *t*-test (values of *P<0.0005). The expression levels of IE1, IE2, and β-actin proteins in cell lysates were determined by immunoblotting with specific antibodies. (C) A hypothetical model showing that expression of IE2 and its SUMOylation regulates the PIAS1-mediated IE1 SUMOylation, enhancing IE1 activity to downregulate type I IFN-stimulated gene (ISG) expression. ISRE, interferon stimulated response element.

We further investigated whether IE2 inhibiting IE1 SUMOylation affects the ability of IE1 to downregulate the promoter containing the IFN stimulated response element (ISRE). In luciferase reporter assays using the ISG54 ISRE-luciferase reporter gene, coexpressing SUMO-1 and PIAS1 inhibited the ability of IE1 to suppress ISRE promoter induction by IFNβ. However, adding IE2(346–542) reversed this effect (Fig. 7A). IE2(346–542) does not contain the transactivation domains (codons 25–85 and 544–579) [55]. Consistently, in a control experiment, IE2(346–542) did not affect the induction of ISRE promoter by IFNβ (Fig. 7B). This result suggests that IE2 expression and its PIAS1-binding activity can interfere with PIAS1-mediated IE1 SUMOylation, resulting in unmodified IE1 more efficiently suppressing type I IFN-mediated ISG expression (Fig. 7C).

## Discussion

In this study, we performed *in silico* analysis to predict possible SUMO modification sites in all HCMV ORFs. Among 24 ORFs that were predicted to have a consensus sequence with relatively high scores, only UL123 (IE2), UL123 (IE1), and US34A, which received the highest scores using the SUMOsp program, were SUMOylated in *E. coli* SUMOylation assays. The SUMOylation levels of US34A in *E. coli* and in cotransfected cells were much lower those of IE1 and IE2, although US34A was SUMOylated as efficiently as IE1 *in vitro*. Unlike IE1 and IE2, the predicted SUMOylation site in US34A was not in the disordered region. Therefore, whether US34A SUMOylation occurs during virus infection needs to be addressed. Given that SUMOylation of IE1 and IE2 was easily detectable in virus-infected cells [20], [39], [40], [44](this study), the data from our *in silico* genome-wide analysis suggest that these two IE proteins may be the main HCMV-encoded targets for SUMO. Nevertheless, we cannot exclude the possibility that SUMOylation of other HCMV proteins predicted in this study occurs in virus-infected cells. An example is UL44. Although we could not detect SUMOylation of UL44 in *E. coli* assays, SUMOylated UL44 was detected in cotransfected cells, *in vitro* SUMOylation reactions, and virus-infected cells [56]. We also observed SUMOylation of UL44 in *in vitro* assays (data not shown).

We and others have found SUMO in viral replication compartments (RCs) in HCMV-infected cells [25], [57], suggesting that viral or cellular SUMO substrates may accumulate at viral RCs. Although IE2 is recruited to viral RCs [58], SUMO is also found in viral RCs in cells infected with a virus encoding a mutant IE2 protein that lacks both the SUMOylation sites and the SIM [57]. Thus, other viral proteins implicated in viral DNA replication have been suggested to be SUMO targets or recruit SUMO via the SIM-mediated intercation. SUMOylation of UL44 (polymerase percessicity factor) might explain the presence of SUMO species in viral RCs. In addition, UL54 (DNA polymerase), UL57 (single-stranded DNA-binding protein), and UL105 (DNA helicase) have been suggested to have SUMO modification sites [57]. Our *in silico* analysis also predicted these viral replication proteins to have possible SUMOylation sites; however, none were SUMOylated in our *E. coli* SUMOylation assays.

In this study, we demonstrated that SUMOylation of IE1 and IE2 is temporally regulated during HCMV infection. SUMOylated IE1 levels were increased at the early phase of infection and decreased at the late phase when the expression of IE2 and its SUMO-modified forms drastically increased. The increase of IE2 SUMOylation at the late stage of infection is consistent with a general role of IE2 SUMOylation in increasing viral gene expression [20]–[24], [26]. The biphasic regulation of IE1 SUMOylation is intriguing. The role of IE1 SUMOylation in viral infection is not clear. A mutant virus encoding SUMOylation-defective IE1 grew less efficiently than normal virus, suggesting a positive role of IE1 SUMOylation in virus infection [41]. However, a similar mutant virus did not have a significant growth defect [42], and the lack of IE1 SUMOylation did not affect the ability of IE1 to complement the growth defect of the IE1-deleted mutant virus [40]. Further studies are necessary to address whether IE1 SUMOylation plays a role at early steps of the viral replication cycle or whether IE1 SUMOylation is just a consequence of IE1 targeting to PML nuclear bodies, where the components of SUMOylation machinery are enriched. Recently, we found that IE1 SUMOylation inhibited the interaction between IE1 and STAT2 and that the SUMO-modified form of IE1 failed to inhibit IFNβ-mediated activation of the ISRE-containing promoter [37]. These findings suggested that IE1 SUMOylation may be detrimental for viruses trying to evade cellular innate immune responses, although the overall effect of IE1 SUMOylation on viral replication could be different. In this regard, IE2, by inhibiting IE1 SUMOylation, may assist in immune escape by the virus. The interplay between SUMOylation of two viral proteins has been shown in Epstein-Barr virus. The SIM-containing BGLF4 protein inhibits BZLF1 SUMOylation through its SUMO-binding activity and also reduces overall SUMOylation, which enhances EBV lytic infection [59], [60].

Several viral proteins have been shown to reduce cellular SUMOylation by directly targeting SUMOylation machinery. The Gam1 protein of avian adenovirus CELO (chicken embryo lethal orphan) reduces cellular SUMOylation by interacting with and destabilizing the SAE1-SAE2 complex [61], [62]. Human papillomavirus E6 induces degradation of Ubc9 [63]. Our finding that IE2 expression inhibits IE1 SUMOylation by binding to PIAS1 raises a question whether IE2 has a general role in regulating the cellular SUMO pathway. We observed that IE2 overexpression slightly reduces the level of cellular SUMO conjugates (data not shown). This intriguing hypothesis remains to be addressed.
